# 依库珠单抗治疗造血干细胞移植相关血栓性微血管病11例临床分析

**DOI:** 10.3760/cma.j.cn121090-20240722-00273

**Published:** 2025-05

**Authors:** 雪宜 罗, 瑞 马, 慧芳 王, 露 柏, 云 何, 圆圆 张, 婷婷 韩, 道兴 邓, 育红 陈, 伟 韩, 晓辉 张, 昱 王, 晓军 黄, 于谦 孙

**Affiliations:** 北京大学人民医院、北京大学血液病研究所、国家血液系统疾病临床医学研究中心，北京 100044 Peking University People's Hospital, Peking University Institute of Hematology, National Clinical Research Center for Hematologic Disease, Beijing 100044, China

**Keywords:** 造血干细胞移植相关血栓性微血管病, 依库珠单抗, 造血干细胞移植, Transplant-associated thrombotic microangiopathy, Eculizumab, Hematopoietic stem cell transplantation

## Abstract

**目的:**

评估依库珠单抗（Eculizumab）治疗造血干细胞移植相关血栓性微血管病（TA-TMA）的疗效。

**方法:**

纳入2018年6月至2024年5月在北京大学人民医院接受异基因造血干细胞移植后发生TA-TMA并应用依库珠单抗治疗的11例患者，对TA-TMA发生情况、治疗及临床结局进行回顾性研究。

**结果:**

纳入研究的11例患者中男4例，女7例，中位年龄29（9～56）岁；重型再生障碍性贫血（SAA）5例、急性淋巴细胞白血病（ALL）3例、急性髓系白血病（AML）3例。TMA中位诊断时间为移植后48（4～213）d，所有患者均符合高危TA-TMA诊断标准。诊断TMA到启动依库珠单抗治疗的中位间隔时间为12（1～56）d，依库珠单抗治疗中位次数为3（1～14）次。10例患者在依库珠单抗治疗结束和死亡时评估疗效均为无反应（NR）。1例曾获得部分缓解（PR），后续因感染引起TA-TMA复发后死亡。截止末次随访，所有患者均失访/死亡，中位随访时间88（33～326）d，诊断TA-TMA至末次随访时间为31（21～113）d。

**结论:**

依库珠单抗治疗TA-TMA疗效较差，可能与患者病情危重、病情复杂、启动依库珠单抗治疗较晚及剂量不足有关。

## Abstract (en)

**Objective:**

To evaluate the efficacy of eculizumab in treating hematopoietic stem cell transplantation-associated thrombotic microangiopathy（TA-TMA）.

**Methods:**

This retrospective study included 11 patients who developed TA-TMA after allogeneic hematopoietic stem cell transplantation and subsequently received eculizumab treatment at Peking University People's Hospital between June 2018 and May 2024. The incidence of TA-TMA, treatment details, and clinical outcomes were analyzed.

**Results:**

Among the 11 included patients［4 males, 7 females; median age: 29 years（range: 9–56）］, underlying diseases were severe aplastic anemia（SAA）in 5 patients, acute lymphoblastic leukemia（ALL）in 3 patients, and acute myeloid leukemia（AML）in 3 patients. The median time to TA-TMA diagnosis was 48 days post-transplantation（range: 4–213 days）, and all patients met the diagnostic criteria for high-risk TA-TMA. The median interval from TA-TMA diagnosis to the initiation of eculizumab treatment was 12 days（range: 1–56 days）. Patients received a median of 3 doses of eculizumab（range: 1–14）. Ten of the 11 patients were assessed as having no response（NR）to eculizumab at the end of treatment or at death. One patient achieved a partial response（PR）but subsequently died after TA-TMA relapsed due to infection. At the last follow-up, all patients were either lost to follow-up or had died. The median follow-up duration was 88 days（range: 33–326 days）, and the median time from TA-TMA diagnosis to the last follow-up was 31 days（range: 21–113 days）.

**Conclusion:**

Eculizumab demonstrated poor efficacy in this TA-TMA cohort. This might be attributable to the critical and complex condition of the patients, delayed initiation of eculizumab treatment, and insufficient dosage.

移植相关血栓性微血管病（TA-TMA）是异基因造血干细胞移植（allo-HSCT）后的严重并发症，病死率可高达50％～90％[1]–[3]。TA-TMA的发病机制尚不完全明确，主要认为与预处理药物、钙调蛋白抑制剂、感染和移植物抗宿主病（GVHD）等引起的血管内皮细胞损伤有关[4]–[5]。依库珠单抗（Eculizumab）是一种人源型抗补体C5单克隆抗体，可通过阻断补体膜攻击复合物（C5b-9）的形成阻止内皮和组织损伤[2],[6]–[9]。依库珠单抗将高危TA-TMA的缓解率提高至93％[10]，长期生存率提高至71％[11]。本研究对本中心既往使用依库珠单抗治疗TA-TMA的情况进行回顾性分析，旨在为该药物的临床应用提供参考。

## 病例与方法

一、病例来源

本项回顾性研究纳入2018年6月至2024年5月期间在北京大学人民医院接受依库珠单抗治疗的allo-HSCT后合并TA-TMA患者11例。

二、研究方法

以检索病历方式获取患者年龄、性别、供受者性别、疾病诊断、人类白细胞抗原（HLA）配型、干细胞来源、预处理方案、GVHD发生、粒细胞及血小板植入时间等临床信息。本研究经北京大学人民医院伦理委员会批准，所有患者均签署知情同意书。

三、移植方案

1. 预处理方案：均接受改良白消安/环磷酰胺（Bu-Cy）联合兔抗人胸腺细胞球蛋白（ATG）预处理方案，部分患者预处理方案包含地西他滨、氟达拉滨或全身放射治疗（TBI）。重型再生障碍性贫血患者采用Cy+ATG方案。所有患者使用前列腺素E1预防肝小静脉闭塞症。

2. GVHD诊断及预防：急性GVHD分度及分级参照改良Glucksberg分度法[12]。采用环孢素A+短程甲氨蝶呤+霉酚酸酯方案进行经典GVHD的预防。

3. 造血重建标准：粒细胞植入定义为连续3 d外周血中性粒细胞绝对计数≥0.5×10^9^/L，血小板植入定义为连续7 d血小板计数≥20×10^9^/L且脱离血小板输注。

4. TA-TMA的诊断：本组病例TA-TMA的诊断参照Jodele标准[8],[11],[13]–[14]。确诊TA-TMA且满足以下3项标准中的≥2项判定为高危TA-TMA：①随机尿蛋白/肌酐比值（rUPCR）≥2 mg/mg；②sC5b-9升高（≥244 µg/L）；③多器官功能障碍综合征（MODS）的临床证据。

四、治疗反应评估

参考文献[11]进行治疗反应评估。完全缓解（CR）：高风险标志物检测结果正常（sC5b-9<244 µg/L，rUPCR<1 mg/mg），14 d内未输注红细胞和血小板，MODS症状消失；部分缓解（PR）：未达到CR标准，但病情有所改善；无反应（NR）：sC5b-9和rUPCR检测未达正常范围、未脱离输血支持、完成治疗后重要器官功能未恢复或死于TA-TMA。

五、随访

以检索病历方式获取随访数据，随访截止时间为2024年7月9日。总生存（OS）时间指从移植至随访结束或死亡时间。

## 结果

一、患者一般情况

11例高危TA-TMA患者中男4例、女7例，中位年龄29（9～56）岁；重型再生障碍性贫血（SAA）5例、急性淋巴细胞白血病（ALL）3例、急性髓系白血病（AML）3例。同胞全相合移植1例，单倍体移植10例，基线特征见表1。

**表1:** 11例接受依库珠单抗治疗造血干细胞移植相关血栓性微血管病患者的一般资料和移植特征

例号	性别	年龄（岁）	诊断	供者类型	预处理方案	干细胞来源	粒细胞植入（d）	血小板植入（d）
1	女	56	ALL	同胞全相合	Bu/Cy+ATG	PB	20	10
2	男	11	SAA	单倍体	Bu/Cy/FLU+ATG	BM+PB	20	未植活
3	男	14	ALL	单倍体	TBI/Cy+ATG	PB	14	12
4	女	15	SAA	单倍体	Bu/Cy+ATG	BM+PB	10	15
5	女	47	AML	单倍体	Bu/Cy+ATG	PB	15	未植活
6	男	44	AML	单倍体	地西他滨+Bu/Cy+ATG	PB	14	77
7	男	28	ALL	单倍体	Bu/Cy+ATG	PB	11	14
8	女	56	SAA	单倍体	Bu/Cy/FLU+ATG	BM+PB	18	未植活
9	女	48	AML	单倍体	地西他滨+Bu/Cy+ATG	PB	10	12
10	女	29	SAA	单倍体	Bu/Cy/FLU+ATG	BM+PB	未植活	未植活
11	女	9	SAA	单倍体	Bu/Cy/FLU+ATG	BM+PB	19	未植活

**注** ALL：急性淋巴细胞白血病；SAA：重型再生障碍性贫血；AML：急性髓系白血病；Bu：白消安；Cy：环磷酰胺；ATG：抗人胸腺细胞球蛋白；FLU：氟达拉滨；TBI：全身放射治疗；PB：外周血；BM：骨髓

二、诊断TA-TMA时的临床特征

中位诊断时间为移植后48（4～213）d，所有患者均符合高危TA-TMA诊断标准，3例患者ADAMTS13<10％，8例患者存在肾脏损害，6例患者需要正压通气（考虑呼吸系统损害），3例患者存在需要干预的心包积液，5例患者有中枢神经系统（CNS）受累表现。2例患者诊断时未应用免疫抑制剂，5例患者起病时合并GVHD，4例患者合并感染。具体见表2。

**表2:** 11例造血干细胞移植相关血栓性微血管病（TA-TMA）患者的临床表现

例号	TA-TMA诊断距移植时间（d）	LDH峰值（U/L）	诊断时sC5b-9（µg/L）	主要临床表现	ADAMTS13<10％	受累器官	GVHD	合并感染
1	48	1 401	273	蛋白尿、高血压、新发血小板减少、新发贫血、微血管病变	是	肺	急性	无
2	17	2 631	665	蛋白尿、高血压、新发血小板减少、新发贫血、微血管病变	无	肾、肺、浆膜、消化道	无	无
3	58	843	323	蛋白尿、高血压、新发血小板减少、新发贫血	是	肾、肺	无	是
4	18	19 437	1 570	蛋白尿、新发血小板减少、新发贫血、微血管病变	无	肾、CNS	无	无
5	41	307	425	蛋白尿、高血压、新发血小板减少、新发贫血、微血管病变	是	肾	急性	无
6	213	1 585	141	蛋白尿、高血压、新发血小板减少、新发贫血、微血管病变	无	肾、肺、CNS	慢性	无
7	48	943	276	蛋白尿、新发贫血、微血管病变	无	肾、消化道	急性	无
8	150	2 590	222	蛋白尿、高血压、新发血小板减少、新发贫血、微血管病变	无	肾、肺	无	是
9	140	460	>2 130	蛋白尿、新发血小板减少、微血管病变	无	CNS	慢性	是
10	4	1 920	未查	蛋白尿、高血压、新发血小板减少、新发贫血、微血管病变	无	肾、肺、浆膜、CNS	无	无
11	76	930	141	蛋白尿、高血压、新发血小板减少、新发贫血、微血管病变	无	浆膜	无	是

**注** sC5b-9：补体膜攻击复合物；ADAMTS13：血管性血友病因子裂解酶；GVHD：移植物抗宿主病；CNS：中枢神经系统

三、依库珠单抗使用情况

9例患者应用钙调磷酸酶抑制剂或雷帕霉素靶蛋白抑制剂，均停药；9例患者进行血浆置换，启动血浆置换的中位时间为5（2～62）d，1例患者应用去纤苷，4例患者应用利妥昔单抗，诊断TA-TMA到启动依库珠单抗治疗的中位间隔时间为12（1～56）d，依库珠单抗中位应用次数为3（1～14）次。1例患者使用2次依库珠单抗后死亡；1例因费用问题使用2次依库珠单抗后停止依库珠单抗治疗；1例在使用依库珠单抗后寒战高热，使用2次后停用；1例患者应用1次后未再应用；2例患者每周使用1次，分别在应用2次及3次后死亡。其余5例患者按照中国专家共识[15]指导用药（起始阶段900 mg每3天1次）。3例在起始剂量阶段死亡，2例进入诱导阶段，其中1例进入维持阶段。10例患者在依库珠单抗治疗前进行sC5b-9检测，中位sC5b-9值为417（141～2 130），其中9例患者sC5b-9高于正常上限。具体见表3。

**表3:** 11例造血干细胞移植相关血栓性微血管病（TMA）患者的依库珠单抗（ECU）治疗情况和疗效判定

例号	ECU启动时间	ECU使用方法	ECU治疗次数	合并治疗	随访时间（d）	疗效
1	13	每3天1次	5	ISM+PTE	32	NR
2	9	每3天1次	2		31	NR
3	23	1次	1	ISM+PTE	25	NR
4	7	每3天1次	4	ISM+PTE+RTX	21	NR
5	5	1次	1	ISM+PTE	21	NR
6	5	每3天1次	11	ISM+PTE+去纤苷	113	NR
7	1	每3天1次	8	PTE+RTX	34	NR
8	23	每周1次	2	ISM+PTE	29	NR
9	20	每3天1次	2		82	NR
10	12	每周1次	3	ISM+PTE+RTX	29	NR
11	56	每3天1次	4	ISM+PTE	91	NR

**注** ISM：停用免疫抑制剂；PTE：血浆置换；RTX：利妥昔单抗；随访时间：诊断TA-TMA到末次随访时间；NR：无反应

四、转归

10例在依库珠单抗治疗结束和死亡时评估疗效均为NR；1例曾获PR，后续因感染引起TA-TMA复发并死亡。截止末次随访，所有患者均失访或死亡，中位随访时间88（33～326）d，诊断TA-TMA至末次随访时间为31（21～113）d。

## 讨论

TA-TMA是移植后严重的合并症，其发病机制尚未阐明，诊断缺乏统一标准，病理诊断是TA-TMA的金标准[15]。近年来研究显示补体异常活化在TA-TMA发病机制中发挥重要作用，利用依库珠单抗阻断C5b-9的形成阻止内皮和组织损伤显著改善了TMA患者的临床预后[2],[6]–[11]。

依库珠单抗治疗TA-TMA的缓解率为50％～93％，OS率为33％～78％[10],[16]–[18]。一项前瞻性临床研究纳入儿童及青少年高危TA-TMA患者，使用依库珠单抗治疗的受试者在高危TA-TMA诊断后6个月的OS率为71％，移植后1年OS率为62％，而未经治疗的历史对照组的OS率为18％[11]。本组病例虽然应用了依库珠单抗，诊断到末次随访的中位时间仅为31（21～113）d，11例患者中2例放弃治疗失访，其余患者均死亡，死亡时TA-TMA疗效评估均为NR，疗效远低于既往报道。分析其原因可能为：①本组患者病情危重，启动依库珠单抗较晚且剂量不足；②患者病情复杂，3例患者ADAMTS13<10％，不能排除血栓性血小板减少性紫癜（TTP）诊断。

首先，启动依库珠单抗治疗较晚可能与疗效不佳有关，Jodele等[11]报告的中位启动时间为诊断后2（2～4）d。Bohl等[10]、Rudoni等[19]、de Fontbrune等[20]报道的中位启动时间分别为10 d、4 d、31 d，缓解率分别为93％、70％、50％，提示依库珠单抗的疗效可能与启动治疗的时机相关。本组病例启动依库珠单抗治疗的中位时间为诊断后12 d，迟于既往研究，可能与疗效不佳有关。

其次，用药次数较少可能与效果不佳互为因果。de Fontbrune等[20]的研究中，依库珠单抗的诱导治疗为900 mg每周1次×4周，随后进入隔周给药1 200 mg维持治疗阶段，依库珠单抗中位治疗次数为6（1～31）次，中位起效时间为6次治疗。Bohl等[10]的治疗方案与de Fontbrune等[20]一致，中位治疗次数为9（1～23）次。Dhakal等[21]对既往报道病例进行Meta分析，认为5.5次依库珠单抗治疗后达到缓解。Jodele等[11]开展的前瞻性研究中，依库珠中位使用次数为20（16～27）次，TA-TMA相关指标恢复正常需要22（13～24）周；使用方法：5次加载剂量（每48 h 2次，每72 h 3次），然后每周1次诱导剂量，共4周，之后隔周1次维持剂量。其之前的研究数据显示，依库珠单抗给药11～13 d后才能达到治疗性谷浓度（≥100 mg/L）并获得预期临床反应，因此，用药<4次的患者被视为不可评估，不纳入研究[22]。

本组患者依库珠单抗的中位使用次数为3（1～14）次，其中1例仅使用1次依库珠单抗后死亡，1例因费用问题使用2次依库珠单抗后停止依库珠治疗，1例在使用依库珠后寒战高热，使用2次后停用，1例患者应用1次后未再应用；2例患者每周使用1次，分别在应用2次及3次后死亡；5例患者按照中国专家共识指导[15]，起始阶段900 mg每3天1次，3例在起始剂量阶段死亡，2例进入诱导阶段，存活时间最长的1例患者进入维持阶段但死于TA-TMA复发。进入维持阶段的患者曾达到TA-TMA部分缓解，其余10例患者失访/死亡时均未到达缓解。因此，本组患者大多在病情危重时才启动依库珠单抗治疗且未能坚持至药物起效，可能是预后差的原因之一。

本组患者中3例ADAMTS13偏低，不能排除TTP诊断，病情更为复杂，按照既往研究标准，通常不纳入依库珠单抗治疗指征，因此也可能是本组患者效果不佳的原因之一。

此外，既往研究所报道的缓解率虽然类似，但是由于时间跨度较大，入组及诊断标准并不完全一致：Jodele等[14]在2020年报道的数据中，根据其提出的诊断标准，64例受试者中有59例在移植后100 d内诊断TA-TMA［中位诊断时间23（3～48）d］，其余5例患者在移植后118 d​～221 d​期间发生晚期TA-TMA。近年来Jodele提出的诊断标准受到更多研究者认可[13]，我们参考Jodele标准对本组病例进行诊断，中位诊断时间为移植后48（4～213）d，3例患者在移植100 d后起病。采用O-TMA标准[23]的研究中，de Fontbrune等[20]报道的中位诊断时间为移植后121（37～455）d，Rudoni等[19]报道中位诊断时间为移植后93（26～334）d，Bohl等[10]报告的中位诊断时间为移植后8.8（2.4～29.5）个月。虽然暂无研究表明早期TA-TMA较晚期TA-TMA更为凶险，但治疗效果不佳与免疫重建不完全的相关性不能排除。

综上所述，本组TA-TMA病例依库珠单抗疗效不佳可能与患者病情危重、启动治疗较晚及剂量不足有关。随着药物可及性改善和治疗方案的优化，依库珠单抗治疗TA-TMA的疗效可能会有所改善。

## References

[1] Sartain S, Shubert S, Wu MF (2019). Therapeutic plasma exchange does not improve renal function in hematopoietic stem cell transplantation-associated thrombotic microangiopathy: an institutional experience[J]. Biol Blood Marrow Transplant.

[2] Jodele S, Laskin BL, Dandoy CE (2015). A new paradigm: Diagnosis and management of HSCT-associated thrombotic microangiopathy as multi-system endothelial injury[J]. Blood Rev.

[3] Elsallabi O, Bhatt VR, Dhakal P (2016). Hematopoietic Stem Cell Transplant-Associated Thrombotic Microangiopathy[J]. Clin Appl Thromb.

[4] Young JA, Pallas CR, Knovich MA (2021). Transplant-associated thrombotic microangiopathy: theoretical considerations and a practical approach to an unrefined diagnosis[J]. Bone Marrow Transplant.

[5] Laskin BL, Goebel J, Davies SM (2011). Small vessels, big trouble in the kidneys and beyond: hematopoietic stem cell transplantation–associated thrombotic microangiopathy[J]. Blood.

[6] Meri S, Bunjes D, Cofiell R (2022). The Role of Complement in HSCT-TMA: Basic Science to Clinical Practice[J]. Adv Ther.

[7] Jodele S, Licht C, Goebel J (2013). Abnormalities in the alternative pathway of complement in children with hematopoietic stem cell transplant-associated thrombotic microangiopathy[J]. Blood.

[8] Jodele S, Davies SM, Lane A (2014). Diagnostic and risk criteria for HSCT-associated thrombotic microangiopathy: a study in children and young adults[J]. Blood.

[9] Rotz SJ, Luebbering N, Dixon BP (2017). In vitro evidence of complement activation in transplantation-associated thrombotic microangiopathy[J]. Blood Adv.

[10] Bohl SR, Kuchenbauer F, Von Harsdorf S (2017). Thrombotic microangiopathy after allogeneic stem cell transplantation: a comparison of eculizumab therapy and conventional therapy[J]. Biol Blood Marrow Transplant.

[11] Jodele S, Dandoy CE, Aguayo-Hiraldo P (2024). A prospective multi-institutional study of eculizumab to treat high-risk stem cell transplantation–associated TMA[J]. Blood.

[12] Przepiorka D, Weisdorf D, Martin P (1995). 1994 Consensus Conference on Acute GVHD Grading[J]. Bone Marrow Transplant.

[13] Schoettler ML, Carreras E, Cho B (2023). Harmonizing Definitions for Diagnostic Criteria and Prognostic Assessment of Transplantation-Associated Thrombotic Microangiopathy: A Report on Behalf of the European Society for Blood and Marrow Transplantation, American Society for Transplantation and Cellular Therapy, Asia-Pacific Blood and Marrow Transplantation Group, and Center for International Blood and Marrow Transplant Research[J]. Transplant Cell Ther.

[14] Jodele S, Dandoy CE, Lane A (2020). Complement blockade for TA-TMA: lessons learned from a large pediatric cohort treated with eculizumab[J]. Blood.

[15] 中华医学会血液学分会造血干细胞应用学组 (2021). 造血干细胞移植相关血栓性微血管病诊断和治疗中国专家共识(2021年版)[J]. 中华血液学杂志.

[16] Jodele S, Fukuda T, Mizuno K (2016). Variable eculizumab clearance requires pharmacodynamic monitoring to optimize therapy for thrombotic microangiopathy after hematopoietic stem cell transplantation[J]. Biol Blood Marrow Transplant.

[17] Vasu S, Wu H, Satoskar A (2016). Eculizumab therapy in adults with allogeneic hematopoietic cell transplant-associated thrombotic microangiopathy[J]. Bone Marrow Transplant.

[18] Jan AS, Hosing C, Aung F (2019). Approaching treatment of transplant‐associated thrombotic Microangiopathy from two directions with Eculizumab and transitioning from Tacrolimus to Sirolimus[J]. Transfusion (Paris).

[19] Rudoni J, Jan A, Hosing C (2018). Eculizumab for transplant‐associated thrombotic microangiopathy in adult allogeneic stem cell transplant recipients[J]. Eur J Haematol.

[20] de Fontbrune FS, Galambrun C, Sirvent A (2015). Use of eculizumab in patients with allogeneic stem cell transplant-associated thrombotic microangiopathy: a study from the SFGM-TC[J]. Transplantation.

[21] Dhakal P, Giri S, Pathak R (2017). Eculizumab in transplant-associated thrombotic microangiopathy[J]. Clin Appl Thromb.

[22] Jodele S, Fukuda T, Vinks A (2014). Eculizumab therapy in children with severe hematopoietic stem cell transplantation-associated thrombotic microangiopathy[J]. Biol Blood Marrow Transplant.

[23] Cho BS, Yahng SA, Lee SE (2010). Validation of recently proposed consensus criteria for thrombotic microangiopathy after allogeneic hematopoietic stem-cell transplantation[J]. Transplantation.

